# Autophagosome-Mediated EGFR Down-Regulation Induced by the CK2 Inhibitor Enhances the Efficacy of EGFR-TKI on EGFR-Mutant Lung Cancer Cells with Resistance by T790M

**DOI:** 10.1371/journal.pone.0114000

**Published:** 2014-12-08

**Authors:** Kwang Sup So, Cheol Hyeon Kim, Jin Kyung Rho, Sun Ye Kim, Yun Jung Choi, Joon Seon Song, Woo Sung Kim, Chang Min Choi, Young Jin Chun, Jae Cheol Lee

**Affiliations:** 1 Department of Pulmonary and Critical Care Medicine, Asan Medical Center, College of Medicine, University of Ulsan, Seoul, Korea; 2 Department of Pathology, Asan Medical Center, College of Medicine, University of Ulsan, Seoul, Korea; 3 Department of Oncology, Asan Medical Center, College of Medicine, University of Ulsan, Seoul, Korea; 4 Asan Institute for Life Sciences, Asan Medical Center, College of Medicine, University of Ulsan, Seoul, Korea; 5 College of Pharmacy, Chung-Ang University, Seoul, Korea; 6 Department of Internal Medicine, Korea Cancer Center Hosptial, Seoul, Korea; University of Manitoba, Canada

## Abstract

Protein kinase CK2 has diverse functions promoting and maintaining cancer phenotypes. We investigated the effect of CK2 inhibition in lung cancer cells with T790M-mediated resistance to the EGFR-TK inhibitor. Resistant sublines of PC-9 to gefitinib (PC-9/GR) and erlotinib (PC-9/ER) were established by previous study, and T790M secondary mutation was found in both resistant sublines. A decrease of EGFR by siRNA treatment effectively controlled the growth of resistant cells, thus suggesting that they still have EGFR-dependency. CX-4945, a potent and selective CK2 inhibitor, induced autophagy in PC-9/GR and PC-9/ER, and which was supported by the induction of autophagic vacuoles and microtubule-associated protein 1 light chain 3 (LC3) expression, and the increase of punctate fluorescent signals in resistant cells pre-transfected with green fluorescent protein (GFP)-tagged LC3. However, the withdrawal of CX-4945 led to the recovery of cancer cells with autophagy. We found that the induction of autophagy by CX-4945 in both resistant cells was CK2 dependent by using small interfering RNA against CK2. The treatment with CX-4945 alone induced a minimal growth inhibition in resistant cells. However, combined treatment of CX-4945 and EGFR-TKI effectively inhibited cancer-cell proliferation and induced apoptosis. CX-4945 increased the translocation of EGFR from the cell surface into the autophagosome, subsequently leading to the decrease of EGFR while inhibition of autophagy by 3MA or Atg7-targeted siRNA pretreatment reduced the decrease of EGFR by CX-4945. Accordingly, apoptosis by a combination of CX-4945 and EGFR-TKI was suppressed by 3MA or Atg7-targeted siRNA pretreatment, thus suggesting that autophagosome-mediated EGFR down-regulation would have an important role regarding apoptotic cell death by EGFR-TKI. Combined treatment of the CK2 inhibitor and EGFR-TKI may be a promising strategy for overcoming T790M-mediated resistance.

## Introduction

Targeting the epidermal growth factor receptor (EGFR) with small-molecule, tyrosine kinase inhibitors has become an essential therapeutic strategy for non-small-cell lung cancer (NSCLC) with EGFR mutation. After confirming the survival benefit compared to that of conventional cytotoxic chemotherapy [1], [2], EGFR-TKIs have been approved as the first-line agents. However, despite the initially remarkable response, acquired resistance eventually develops, thus limiting the median response duration to less than one year [3], [4]. Approximately half of the resistance is caused by a second-site mutation at position 790, namely T790M [5], [6]. The bulkier methionine residue in T790M could hinder the binding of the drug or the increased ATP affinity at the ATP-binding pocket, and thus minimizing the drug efficacy [5], [7].

Second generation EGFR-TKIs, such as BIBW2992 (afatinib) and PF00299804 (dacomitinib), have been recommended in order to overcome the T790M-mediated resistance considering that these potent, irreversible EGFR-TKIs no longer compete with ATP once they have become covalently bound to the kinase domain [8], [9]. However, it is uncertain whether irreversible EGFR-TKIs can overcome the resistance caused by T790M as some preliminary results of on-going clinical trials have been rather disappointing in terms of overcoming the resistance, although more successful, progression-free patient survival could be achieved when used as the first-line agent compared to reversible EGFR-TKIs [10], [11]. Therefore, further clinical investigation will be required in order to provide more effective overcoming strategies.

Protein kinase CK2 is a constitutively active and highly conserved, ubiquitous serine/threonine kinase which is involved in a variety of cell signaling related to the cell cycle, proliferation, and apoptosis [12]–[14]. Aberrant CK2 expression and activity have been reported in many human diseases, including cancer [15]. The overexpression of CK2 attenuates the apoptosis of cancer cells, while its down-regulation enhances cell death caused by drug or radiation, and thus suggesting its important regulatory role regarding determination of the cancer-cell fate [16]–[19]. CK2-dependent phosphorylation of Cdc37 is required for the chaperoning function of Hsp90 on numerous client oncoproteins, including CK2, itself [20]. Because Hsp90 is essential for oncoprotein maturation and stability, the survival of cancer cells is critically dependent on its proper function, thus suggesting that the control of HSP90 directly or indirectly through the inhibition of CK2 would be promising for cancer treatment. In addition, CK2 can regulate EGFR and its downstream signaling, especially the activity of members of the PI3K-Akt-mTOR pathway [21]–[24]. The inhibition of this pathway has been shown to potentiate the effect of EGFR inhibitors [25].

In this study, we investigated the activity of CX-4945, a selective and potent CX-2 inhibitor, on EGFR-mutant lung cancer cells with T790M mutation leading to resistance to EGFR-TKIs. It was also examined whether it could enhance the effect of EGFR-TKIs in order to overcome the resistance.

## Materials and Methods

### Cell culture and reagents

Gefitinib/Erlotinib-resistant cell lines (PC-9/GR and PC-9/ER) were established in a previous study [26]. Cells were cultured in RPMI 1640 (Invitrogen, Carlsbad, CA) containing 10% fetal bovine serum, 100 units/mL penicillin, and 100 µg/mL streptomycin (Invitrogen, Carlsbad, CA) at 37°C in a 5% CO2 atmosphere. The MTT solution was purchased from Sigma (St Louis, MO, USA). Gefitinib, Erlotinib, 17-DMAG, CX-4945, and 3MA were purchased from Selleck Chemicals Co. Ltd (Houston, TX, USA).

### Cell survival assays

To perform the MTT assay, cells were plated in 96-well sterile plastic plates. Cells were exposed to varying doses of CX-4945. After 72 h, 15 µL of MTT solution (5 mg/mL) was added to each well and plates were incubated for 4 h. Crystalline formazan was solubilized with 100 µL of a 10% (w/v) SDS solution for 24 h. Absorbance at 595 nm was read spectrophotometrically using a microplate reader. To validate the combined effects of CX-4945 or EGFR dependency, cells were treated with CX-4945, EGFR-TKIs, a combination of CX-4945 and gefitinib or erlotinib, or EGFR targeted siRNA for the indicated times. Cell viability was determined using an ADAM-MC automatic cell counter (NanoEnTek, Seoul, Korea) according to the manufacturer's instructions.

### Western blot analysis

Whole cell lysates were prepared using EBC lysis buffer (50 mM Tris-HCl [pH 8.0], 120 mM NaCl, 1% Triton X-100, 1 mM EDTA, 1 mM EGTA, 0.3 mM phenylmethylsulfonyl fluoride, 0.2 mM sodium orthovanadate, 0.5% NP40, and 5 U/mL aprotinin) and were then centrifuged. The resulting supernatant (20 µg) was separated on 8% to 12% SDS-PAGE and transferred to PVDF membranes (Invitrogen). The membranes were blocked using 5% skim milk-PBS-0.1% Tween 20 for one hour at room temperature before being incubated overnight with primary antibodies specific for p-CK2α which was purchased from Sigma (St Louis, MO, USA) and CK2α which was purchased from Abcam (Cambridge, UK). p-EGFR (Tyr1173), EGFR, Caspase-3, Akt, p-Erk, Erk, and β-actin were obtained from Santa Cruz Biotechnology (Santa Cruz, CA, USA). P-Akt (Ser473), cleaved PARP (Asp214), Atg7 and LC3 were purchased from Cell Signaling Technology (Berverly, MA, USA). Horseradish peroxidase–conjugated antibodies were used as secondary antibodies. Membranes were developed using ECL kits (PerkinElmer, Waltham, MA, USA).

### Acridine orange staining

Autophagy was analyzed by staining cells with the vital dye, acridine orange (Sigma, St Louis, MO). Cells were trypsinized and were incubated with acridine orange at a final concentration of 5 µg/mL for 30 min at 37°C in a 5% CO2 atmosphere. Analyses were performed on a FACScan (Becton Dickinson, Franklin Lakes, NJ, USA). The data were analyzed using CellQuest software (Becton Dickinson). The results are representative of at least three, independent experiments, and the error bars signify standard deviations (SDs).

### LC3-GFP expression

Cells were transfected with a plasmid, pEGFP-LC3 vector (Addgene Inc., Cambridge, MA, USA) and were cultured for 24 h. Cells were then treated with CX-4945 for 24 h. The punctate patterns of LC3 in transfected cells were examined by fluorescence microscopy.

### Apoptosis assay

Apoptosis was quantified using the Annexin V-FITC apoptosis kit (BD Biosciences, San Diego, CA, USA) in accordance with the manufacturer's instructions. In brief, cells were trypsinized, pelleted by centrifugation, and resuspended in Annexin V binding buffer (150 mM NaCl, 18 mM CaCl_2_, 10 nM HEPES, 5 mM KCl, 1 mM MgCl_2_). FITC-conjugated Annexin V (1 µg/ml) and propidium iodide (50 µg/ml) was added to the cells which were incubated for 30 min at room temperature in the dark. Analyses were done on a FACScan (Becton Dickinson). The data were analyzed using CellQuest software (Becton Dickinson). The results are representative of at least three, independent experiments, and the error bars signify standard deviations (SDs).

### Small interfering RNA transfection

Small interfering RNA (siRNA) oligonucleotides specific for EGFR, Atg7, CK2α and the siRNA control were purchased from Santa Cruz Biotechnology (Santa Cruz). Cells were seeded into a 60-mm dish which was then left for 24 h. A 2 µL aliquot of siRNA solution (10 µM) and 5 µL of Lipofectamine 2000 (Invitrogen) were each mixed with 100 µL of serum-free RPMI 1640 medium. They were incubated for 20 min at room temperature after combining the two mixtures, and this was then added to the cells that had been seeded on the dish. After 24 h, harvested cells were subjected to Western blot analysis. Cells were also processed for cell viability and apoptosis analysis.

### Confocal microscopy

The cells were grown in a chamber slide in the presence or absence of CX-4945 for 48 h. They were fixed for 10 min with cold methanol and then washed three times with PBS. The cells were incubated with anti-EGFR (Santa Cruz, 1∶50) and LC3 (Cell signaling, 1∶100) overnight at 4°C. Secondary antibody incubation included 1∶100 dilution of either Alexa Fluor 488 anti-mouse or Alexa Fluor 568 anti-rabbit antibodies. Nuclei were stained with 4′,6-diamidino-2-phenylindole (DAPI) and were then washed and cover-slipped. Slides were viewed under an LSM710 confocal laser scanning microscope (Carl Zeiss, Jena, Germany), and the images were photographed.

## Results

### CX-4945 induces autophagy in gefitinib/erlotinib-resistant PC-9 cells

**Figure 1:**
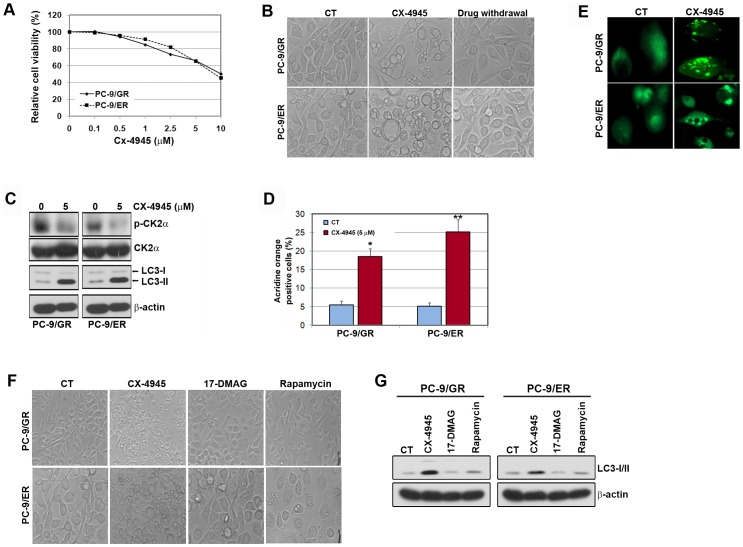
CX-4945 induced the autophagy in EGFR-TKI-resistant PC-9 cells. A, Cells were treated with different concentrations of CX-4945 for 72 h, and the rate of inhibition was determined by MTT assay. B, Cells were treated with or without CX-4945 (5 µM) for 48 h and were then incubated for 24 h with a drug-free medium containing 10% FBS. Pictures showing the autophagic vacuole formation (AVOs) were taken at ×20 magnification. C and D, Cells were treated with CX-4945 for 48 h. Cell lysates were subjected to Western blot analysis. Quantitative detection of acidic vesicular organelles by acridine orange staining of cells was determined by FACS analysis. *p<0.01 and **p<0.001 compared with the control. E, Cells were transfected with a plasmid to express LC3-GFP. After 24 h transfection, cells were treated with CX-4945 (5 µM) for 24 h. Punctate pattern of LC3 localization analyzed by immunofluorescence microscopy. F and G, Cells were incubated with CX-4945 (5 µM), 17-DMAG (100 nM) or rapamycin (20 µM) for 48 h. Pictures were taken at ×20 magnification. The induction of LC3-I/II was shown by Western blot analysis.

Some studies have reported that CX-4945 led to an inhibition of proliferation in human cancer cells [15], [24]. To investigate whether CX-4945 can inhibit the growth of lung cancer cells with T790M-mediated resistance to EGFR-TKIs, cells were treated with CX-4945 in a dose-dependent manner. As shown in Fig. 1A, CX-4945 treatment did not show a significant growth inhibition in gefitinib/erlotinib-resistant cells. However, we found that CX-4945 treatment triggered the accumulation of autophagic vacuoles (AV), whereas these cells were restored to their previous condition after drug withdrawal (Fig. 1B). To further confirm the induction of autophagy by CX-4945, we analyzed the expression of the autophagy marker, LC3-II, and the number of cells stained with acridine orange by performing Western blotting and FACS analysis. Consistent with the induction of autophagic vaculoles, CX-4945 treatment showed a strong increase in the amount of endogenous LC3-II and in the percentage of acridine orange-positive cells (Fig. 1C and D). In addition, CX-4945 treatment exhibited characteristic punctate pattern of LC3, whereas the vehicle-treated cells showed diffuse and weak LC3-associated green fluorescence (Fig. E). This CX-4945-induced autophagy was also observed in parental PC-9 cells (data not shown). These results demonstrated that CX-4945 has the capability to trigger the induction of autophagy in parental cells as well as gefitinib/erlotinib-resistant PC-9 cells.

Hsp90 is essential for numerous oncoproteins maturation and stability including CK2α [20]. In addition, the chaperoning function of Hsp90 required phosphorylation of Cdc37 by CK2α [27], [28]. To further evaluate whether Hsp90 was involved in CX-4945-induced autophagy, cells were treated with 17-DMAG, Hsp90 inhibitor. As shown in Fig. 1F and G, the inhibition of Hsp90 did not show the induction of autophagy. Although cells were treated with the same doses of each drug, CX-4945-induced autophagy was more potent than rapamycin, an mTOR inhibitor.

**Figure 2:**
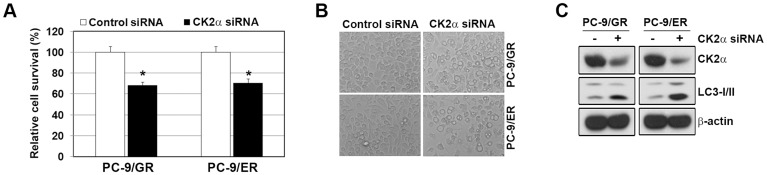
Down-regulation of CK2α by siRNA treatment induced the autophagy in EGFR-TKI-resistant PC-9 cells. A and B, Cells were transfected with control or CK2α siRNA (100 nM) for 48 h. Cell numbers were determined with an ADAM-MC automatic cell counter. Pictures showing the autophagic vacuole formation (AVOs) were taken at ×20 magnification. *p<0.01 compared with the control. C, After 48 h transfection, CK2α and LC3-I/II was shown by Western blot analysis.

Although CX-4945 has the selectivity for CK2, it can inhibit other kinases, such as FLT3, PIM1, and CDK1. Thus, we examined whether CX-4945-induced autophagy is dependent on CK-2. Consistent with the result of CX-4945 treatment, the suppression of CK2α induced only a minimal growth inhibition, whereas it resulted in the increase of autophagic vaculoles and endogenous LC3-II (Fig. 2). These result suggested that CX-4945-induced autophagy might be CK2-dependent.

### CX-4945 enhances the efficacy of EGFR-TKIs in gefitinib/erlotinib-resistant cells

**Figure 3:**
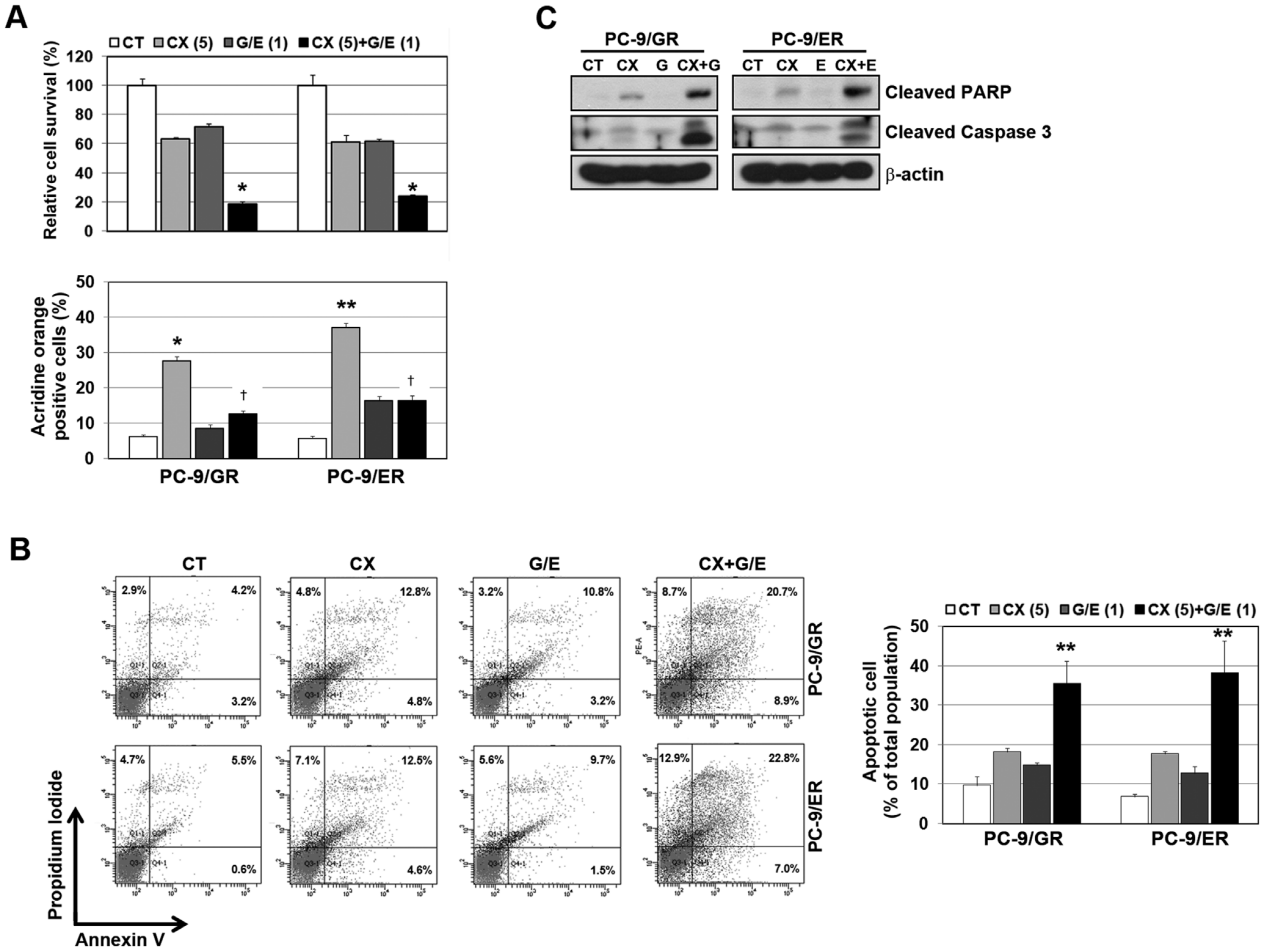
CX-4945 enhanced the efficacy of EGFR-TKIs to overcome drug resistance caused by T790M. Cells were treated with CX-4945 (5 µM), gefitinib (1 µM), and erlotinib (1 µM) or a combination of CX-4945 and gefitinib or CX-4945 and erlotinib for 48 h. A, Cell numbers were determined using a cell counter (upper panel). Quantitative detection of acidic vesicular organelles by acridine orange staining of cells was determined by FACS analysis (lower panel). †p<0.01 compared with CX-4945 alone. B, Apoptosis was assessed by Annexin V-FITC/Propidium iodide staining and flow cytometry. Diagrams of Annexin V-FITC/Propidium iodide flow cytometry in a representative experiment are presented at the left panel. The results are representative of at least 3 independent experiments, and the error bars signify standard deviations (±SDs). C, Cleavage of PARP-1 and caspase-3 was shown by Western blot analysis. *p<0.01 and **p<0.001 compared with the control.

The relationship between the induction of autophagy and the sensitivity to EGFR-TKIs has controversially remained [29]–[32]. Determining whether CX-4945-induced autophagy can affect the sensitivity to EGFR-TKIs, cells were treated with CX-4945, EGFR-TKI or a combination of both for 48 h. The combination of CX-4945 and gefitinib or erlotinib led to significant growth inhibition, whereas CX-4945-induced autophagy was decreased in combined treatment (Fig. 3A). Furthermore, combined treatment with EGFR-TKIs and CX-4945 induced caspase-3 and PARP-1 cleavage, thus leading to enhanced cell death (Fig. 3B and C).

**Figure 4:**
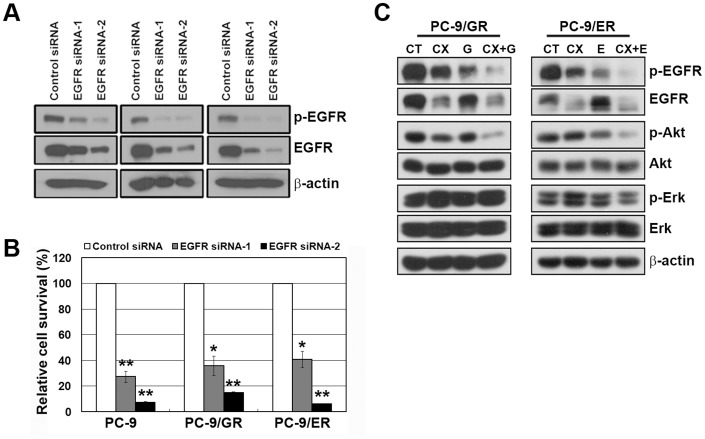
Addition of CX-4945 to EGFR-TKIs substantially suppressed the EGFR signaling pathway in gefitinib/erlotinib-resistant, PC-9 cells. A and B, Control and EGFR siRNAs (100 nM) were introduced into parental or resistant cells, and EGFR suppression was confirmed by Western blot analysis. Cell viability was measured using a cell counter 72 h later. *p<0.01 and **p<0.001 compared with the control. C, Cells were treated with drugs as in Fig. 2. Cells were harvested, and the modulation of EGFR signaling in the indicated cell lines was detected by Western blot analysis.

To investigate the mechanism by which CX-4945 restored the antitumor activities of EGFR-TKI in resistant cells, we firstly examined the dependency of EGFR signaling in both types of resistant cells. Although the efficacy to down-regulate EGFR differed, the growth of both resistant cells was significantly inhibited by siRNA treatment, and thus suggesting the persistence of EGFR dependency despite T790M-mediated resistance (Fig. 4A and B). We next observed the activities of EGFR and its downstream molecules when cells were exposed to each drug. The inhibitory effect of a single treatment with CX-4945, gefitinib or erlotinib on EGFR and Akt activities was modest, whereas the combination of CX-4945 and gefitinib or erlotinib completely suppressed EGFR and Akt activity (Fig. 4C). Treatment with CX-4945 induced the down-regulation of the total EGFR as seen in siRNA treatment. These results suggest that the addition of CX-4945 to EGFR-TKI may overcome T790M-mediated resistance through the increased activity of EGFR-TKI to suppress EGFR signals by the down-regulation of EGFR.

### The inhibition of CX-4945-induced autophagy attenuates apoptosis in gefitinib/erlotinib-resistant cells

**Figure 5:**
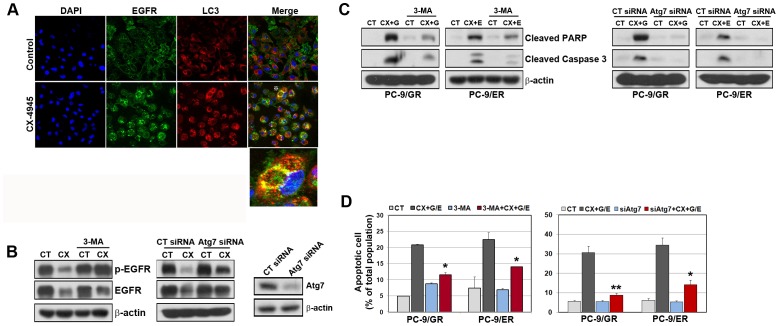
The inhibition of CX-4945-induced autophagy led to decreased apoptosis. A, PC-9/ER cells were treated with CX-4945 (5 µM) for 48 h and then were fixed with methanol, immunostained with anti-LC3 (red), anti-EGFR (green), and DAPI (blue), and analyzed by confocal microscopy to determine the intracellular localization of EGFR. B, The suppression of Atg7 by siRNA treatment was detected by Western blot analysis. PC-9/ER cells were treated with CX-4945 (5 µM) for 48 h in the presence or absence of 3MA (2 mM) and Atg7 siRNA (100 nM). The modulation of EGFR was detected by Western blot analysis. C and D, Cells were treated with drugs as in Fig. 2 under the presence or absence of 3MA and Atg7 siRNA. Cleavage of PARP-1 and caspase-3 was shown by Western blot analysis. Apoptosis was assessed by Annexin V-FITC/Propidium iodide staining and flow cytometry. The results are representative of at least 3 independent experiments, and the error bars signify standard deviations (±SDs). *p<0.01 and **p<0.001 compared with the combination of CX-4945 and gefitinib or erlotinib.

We observed that CX-4945 treatment led to the down-regulation of EGFR. To determine whether the induction of autophagy is associated with the down-regulation of EGFR, the changes in EGFR localization were confirmed by confocal microscopy using the anti-EGFR and anti-LC3 antibody conjugated to fluorophore. As shown in Fig. 5A, CX-4945 treatment showed an increase inLC3/EGFR co-localization. These results demonstrate that CX-4945-induced autophagy can entrap EGFR from plasma membrane into the autophagosome and that trapped proteins may be degraded by lysosome.

To further investigate whether CX-4945-induced autophagy leads directly to the down-regulation of EGFR, we evaluated the level of EGFR in the presence or absence of the autophagic inhibitor (3-methyladenine, 3MA) and Atg7 siRNA treatment. The number of autophagosomes was significantly lower in pre-treated cells with 3MA (data not shown), and 3MA or Atg7 siRNA treatment restored the activities as well as the total level of EGFR (Fig. 5B). Furthermore, the inhibition of autophagy by 3MA or suppression of Atg7 decreased caspase-3 and PARP-1 cleavage and consequently led to the reduction of apoptotic cell death (Fig. 5C and D). Taken together, these results indicate that the induction of autophagy by CX-4945 may have an important role in overcoming T790M-mediated resistance to EGFR-TKIs.

## Discussion

Investigations to provide effective strategies for overcoming T790M-mediated resistance are very important as most of the patients treated with EGFR-TKIs acquired resistance and T790M was the cause of resistance in half of these patients [5], [6]. As the second-generation, irreversible EGFR-TKIs, such as afatinib and dacomitinib, failed to overcome the resistance caused by T790M [33], [34], other measures, such as EGFR dual targeting with cetuximab and afatinib [35] and third-geneneration, mutant-selective EGFR-TKIs [36]–[38] are being actively evaluated. The preliminary reports regarding the efficacy of mutant-selective EGF-TKIs are quite promising [39]. These novel drugs are expected to be clinically available in the near future for patients with T790M-mediated resistance. However, due to tumor heterogeneity and genomic instability, the emergence of resistance to these drugs seems to be unavoidable requiring other therapeutic strategies.

Chen et al. demonstrated that EGFR-specific siRNAs strongly inhibited cell growth and induced apoptosis in H1975 cells harboring both L858R and T790M [40]. Even knock-down of the T790M transcript by siRNAs, when combined with afatinib, was efficacious in controlling T790M-mutant, lung cancer cells. In line with this, we also confirmed the persistence of EGFR dependency in T790M-mutant, lung cancer cells. This is theoretically plausible considering that T790M only reduces the biding affinity of EGFR-TKIs, but does not activate the redundant mechanism for cancer-cell survival except in rare cases having other concomitant, resistant mechanisms. Therefore, the down-regulation of EGFR could exert anti-cancer effects on cancer cells with EGFR dependency as well as enhancing the efficacy of EGFR-TKIs in the setting of a decreased level of EGFR expression. Our study firstly demonstrated that EGFR down-regulation caused by CX-4945 enhanced the efficacy of EGFR-TKI on EGFR-mutant lung cancer cells with T790M-mediated resistance.

Until now, it has been controversially remained whether autophagy is associated with sensitivity or resistance to EGFR-TKIs. However, some papers recently suggested that the induction of autophagy is necessary for the cytotoxic effect of EGFR-TKIs in primary and resistant cells with mutant EGFR [29], [32], [41]. They also showed that enhanced autophagy is required for survival in EGFR-independent EGFR-mutant lung cancer cell [41]. In our study, both resistant cells still had EGFR-dependency and the inhibition of autophagy led to the reduction of cell death. Consistent with previous studies, our results showed that the induction of autophagy has an important role to overcome acquired resistance to EGFR-TKIs although the mechanisms to induce autophagy may be different.

**Figure 6:**
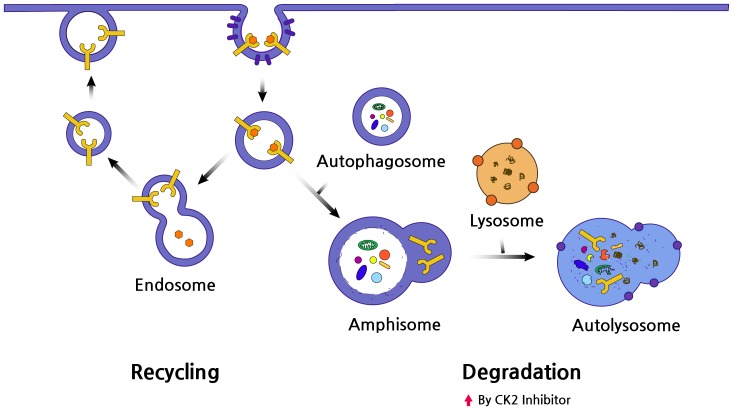
Illustration of EGFR degradation by CX-4945-induced authophagy. EGFR is delivered from the plasma membrane to early endosomes in endocytic vesicles. These vesicles are back to the plasma membrane through the recycling pathway. However, vesicles can also fuse with authophagosomes, and then directly with lysosomes leading to degradation of EGFR.

EGFR expression at the cell surface is tightly controlled by a complex, endocytic mechanism [42]. After the ligand-mediated activation and internalization, EGFR is either recycled back to the cell surface or transported for lysosomal degradation [42]–[44]. Alteration of this delicate balance can change the level of EGFR at the cell surface. Increased autophagosomes in cytoplasm could fuse with endosomes containing EGFR, and thus causing the formation of amphisomes. Subsequently, autolysomes developed by the fusion with lysosome might lead to EGFR degradation (Fig. 6). In our study, CX-4945 induced autophagosomes more potently than rapamycin, a well-known mTOR inhibitor in EGFR-mutant, lung cancer cells with T790M. Decreased EGFR expression in these cells by CX-4945 would be caused by the process mentioned previously. This is supported by results showing that increased internalized EGFR at autophagosomes by CX-4945 could be visualized in fluorescent cytochemical staining and the inhibition of autophagy by 3MA or Atg7 siRNA treatment restored the EGFR level. Therefore, induction of autophagy leading to increased EGFR degradation, when combined with EGFR-TKIs, could be one of the promising therapeutic options for EGFR TKI-resistant cancer cells with EGFR dependency.

CX-4945-induced autophagy may not be mediated by inhibition of chaperoning function of Hsp90 because 17-DMAG could not induce autophagy. Previous studies showed that suppression of CK2 induces autophagic cell death through modulation of the mTOR and MAPK signaling in human glioblastoma cells [45]. In line with this, we found that downregulation or inhibition of CK2α could lead to induction of autophagy in lung cancer cells with EGFR mutation. However, cell death did not occur when resistant cells were treated with CX-4945 or CK2α siRNA. This result might be caused by the characteristic of EGFR-mutant cancer cells. Downstream signalings (PI3K/AKT and MAPK) of cells with EGFR mutation are highly dependent on EGFR activity. Thus, the inhibition of only CK2 may not be sufficient for the modulation of the mTOR and MAPK signaling in these cells. Further detailed mechanisms should be explored by following studies.

Nevertheless, we suggested that CX-4945 could be useful for treatment of EGFR-mutant lung cancer with T790M-mediated resistance. However, for its clinical application, the safety issue related with this novel drug seems to be solved because it could inhibit the function of Hsp90 which is important in maturation and stability of both normal proteins and oncoproteins. Hence, it is uncertain whether it can reach effective serum concentration without significant adverse effects considering that we mostly used 5uM of CX-4945 in our study. Therefore, further investigations are required to address the appropriate dose of CX-4945 and safety profiles.

In summary, autophagosome-mediated EGFR down-regulation induced by CX-4945, a potent and selective CK2 inhibitor, enhances the efficacy of EGFR-TKI on EGFR-mutant lung-cancer cells with resistance by T790M.
