# Expression of Concern: Autophagosome-Mediated EGFR Down-Regulation Induced by the CK2 Inhibitor Enhances the Efficacy of EGFR-TKI on EGFR-Mutant Lung Cancer Cells with Resistance by T790M

**DOI:** 10.1371/journal.pone.0213989

**Published:** 2019-03-14

**Authors:** The PLOS ONE Editors

The authors have informed the *PLOS ONE* Editors that there are image duplication errors in the published paper [1]. Specifically,

- In Fig 1C, the actin bands are duplicated in the PC-9/GR and PC-9/ER panels.
- In Fig 1B and 1F, the images of cellular morphology are the same in the PC-9/ER CT panels.

The authors have provided a corrected version of Fig 1 here.

**Fig 1:**
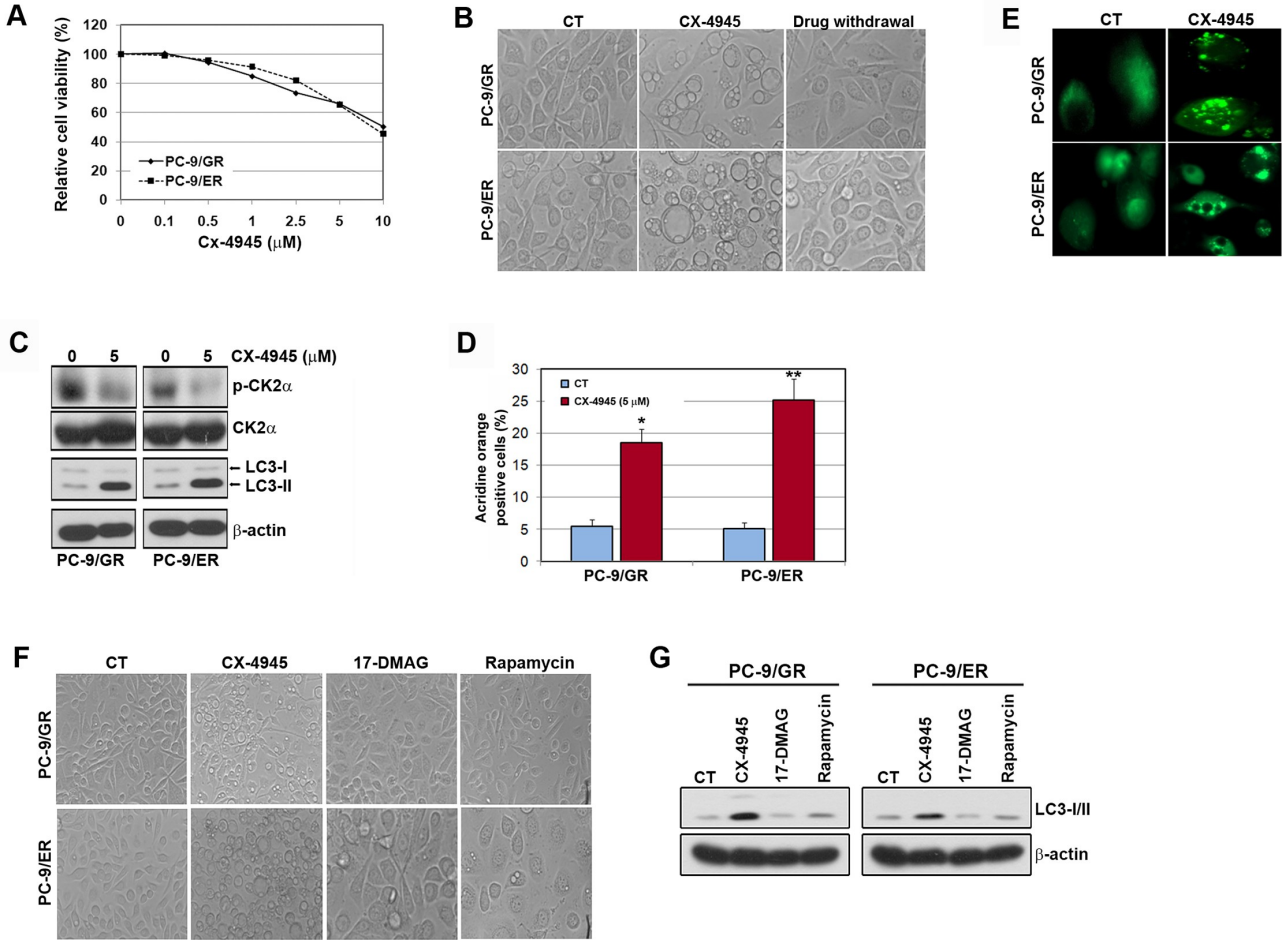
CX-4945 induced the autophagy in EGFR-TKI-resistant PC-9 cells. A, Cells were treated with different concentrations of CX-4945 for 72 h, and the rate of inhibition was determined by MTT assay. B, Cells were treated with or without CX-4945 (5 μM) for 48 h and were then incubated for 24 h with a drug-free medium containing 10% FBS. Pictures showing the autophagic vacuole formation (AVOs) were taken at ×20 magnification. C and D, Cells were treated with CX-4945 for 48 h. Cell lysates were subjected to Western blot analysis. Quantitative detection of acidic vesicular organelles by acridine orange staining of cells was determined by FACS analysis. *p<0.01 and **p<0.001 compared with the control. E, Cells were transfected with a plasmid to express LC3-GFP. After 24 h transfection, cells were treated with CX-4945 (5 μM) for 24 h. Punctate pattern of LC3 localization analyzed by immunofluorescence microscopy. F and G, Cells were incubated with CX-4945 (5 μM), 17-DMAG (100 nM) or rapamycin (20 μM) for 48 h. Pictures were taken at ×20 magnification. The induction of LC3-I/II was shown by Western blot analysis.

The original, unaltered, uncropped image files underlying Fig 1C are no longer available. The replacement image provided in the revised figure was available only as a cropped, processed image from an earlier stage of figure preparation.

Additional Western blot methodological information is provided by the authors as follows: “We sliced blots horizontally using pre-stained markers and stained each separate blot with different primary antibodies. Also, we confirmed actin on the same gel accompanying target proteins. To validate phosphor-form and total form of proteins, western blot membranes were stripped and then re-probed according to protocol (Sigma-Aldrich). We put all figures in PowerPoint before we began making figures of the manuscripts in Photoshop. When we made the figures, we adjusted the blot images to have similar size. If any blot image was captured with larger size than others in PowerPoint, we adjusted the thickness and length of the protein bands to have similar size in Photoshop. However, we did not adjust the contrast and definition of the figures.”

Clarifications and corrections are required to the reporting of the data analysis for the apoptosis assays. Student’s t test was used to compare the means for the reported apoptosis assays. The Methods section for the apoptosis assay and the figure captions for Figs 3 and 5 incorrectly state that the results are representative of at least three independent experiments. The correct sample size for all apoptosis assays presented in the charts in the paper is n = 2.

The available underlying image and data files for this study are provided as Supporting Information files. They can be viewed below.

The *PLOS ONE* Data Availability Policy requires that, with few exceptions, all data underlying the findings described in an article are fully available without restriction.

The Data Availability statement for this article [1] states: “… all data underlying the findings are fully available without restriction. All relevant data are within the paper.”

While looking into the image issues raised by the authors, it came to the attention of the *PLOS ONE* Editors that some of the data used in this study cannot be made available in accordance with the above policy. The original flow cytometry data and replicate dot plots underlying the apoptosis data charts are unavailable. The individual-level data points underlying the charts in Figs 3A and 3B are also unavailable.

The *PLOS ONE* Editors issue this Expression of Concern to alert readers of these concerns about the unavailability of a number of underlying data files and the consequent inability to verify the reliability and accuracy of the Western blot data in Fig 1C following discovery of a duplicate image panel.

## Supporting information

S1 File Underlying images and data. (PDF)
