# Supplementary material for: Expression of Concern: Autophagosome-Mediated EGFR Down-Regulation Induced by the CK2 Inhibitor Enhances the Efficacy of EGFR-TKI on EGFR-Mutant Lung Cancer Cells with Resistance by T790M
Source: PLoS One. 2019 Mar 14;14(3):e0213989. doi: 10.1371/journal.pone.0213989 (PMC6417781; doi:10.1371/journal.pone.0213989)

PLOS ONE

RESEARCH ARTICLE

# Autophagosome-Mediated EGFR Down-Regulation Induced by the CK2 Inhibitor Enhances the Efficacy of EGFR-TKI on EGFR-Mutant Lung Cancer Cells with Resistance by T790M

**Kwang Sup So[1,5⁋], Cheol Hyeon Kim[6⁋], Jin Kyung Rho[1,4], Sun Ye Kim[1], Yun Jung Choi[1], Joon Seon Song[2], Woo Sung Kim[1], Chang Min Choi[1,3], Young Jin Chun[5*], Jae Cheol Lee[3*]**

**1.** Department of Pulmonary and Critical Care Medicine, Asan Medical Center, College of Medicine, University of Ulsan, Seoul, Korea, **2.** Department of Pathology, Asan Medical Center, College of Medicine, University of Ulsan, Seoul, Korea, **3.** Department of Oncology, Asan Medical Center, College of Medicine, University of Ulsan, Seoul, Korea, **4.** Asan Institute for Life Sciences, Asan Medical Center, College of Medicine, University of Ulsan, Seoul, Korea, **5.** College of Pharmacy, Chung-Ang University, Seoul, Korea, **6.** Department of Internal Medicine, Korea Cancer Center Hospital, Seoul, Korea

*jclee@amc.seoul.kr (JCL); yjchun@cau.ac.kr (YJC)

⁋ These authors contributed equally to this work.

OPEN ACCESS

**Citation:** So KS, Kim CH, Rho JK, Kim SY, Choi YJ, et al. (2014) Autophagosome-Mediated EGFR Down-Regulation Induced by the CK2 Inhibitor Enhances the Efficacy of EGFR-TKI on EGFR-Mutant Lung Cancer Cells with Resistance by T790M. PLoS ONE 9(12): e114000. doi:10.1371/journal.pone.0114000

**Editor:** Spencer B. Gibson, University of Manitoba, Canada

**Received:** August 2, 2014

**Accepted:** November 2, 2014

**Published:** December 8, 2014

**Copyright:** © 2014 So et al. This is an open-access article distributed under the terms of the Creative Commons Attribution License, which permits unrestricted use, distribution, and reproduction in any medium, provided the original author and source are credited.

**Data Availability:** The authors confirm that all data underlying the findings are fully available without restriction. All relevant data are within the paper.

**Funding:** This work was funded by a grant of the Korean Health Technology R&D Project, Ministry of Health & Welfare (HI12C1146000013) and a grant (2011-0529) from Asan Institute for Life Science, Seoul, Republic of Korea. The funders had no role in study design, data collection and analysis, decision to publish, or preparation of the manuscript.

**Competing Interests:** The authors have declared that no competing interests exist.

## Abstract

Protein kinase CK2 has diverse functions promoting and maintaining cancer phenotypes. We investigated the effect of CK2 inhibition in lung cancer cells with T790M-mediated resistance to the EGFR-TK inhibitor. Resistant sublines of PC-9 to gefitinib (PC-9/GR) and erlotinib (PC-9/ER) were established by previous study, and T790M secondary mutation was found in both resistant sublines. A decrease of EGFR by siRNA treatment effectively controlled the growth of resistant cells, thus suggesting that they still have EGFR-dependency. CX-4945, a potent and selective CK2 inhibitor, induced autophagy in PC-9/GR and PC-9/ER, and which was supported by the induction of autophagic vacuoles and microtubule-associated protein 1 light chain 3 (LC3) expression, and the increase of punctate fluorescent signals in resistant cells pre-transfected with green fluorescent protein (GFP)-tagged LC3. However, the withdrawal of CX-4945 led to the recovery of cancer cells with autophagy. We found that the induction of autophagy by CX-4945 in both resistant cells was CK2 dependent by using small interfering RNA against CK2. The treatment with CX-4945 alone induced a minimal growth inhibition in resistant cells. However, combined treatment of CX-4945 and EGFR-TKI effectively inhibited cancer-cell proliferation and induced apoptosis. CX-4945 increased the

Figure 1

A

| | | | | | | | |
|---|---|---|---|---|---|---|---|
| 130521 | | | | | | | |
| PC-9, PC-9/GR(L), ER(L) CX4945 only (serum 2%) 9x10³cells/96well | | | | | | | |
| | | | | | | | |
| | CX-4945 (uM) | | | | | | |
| | 0 | 0.1 | 0.5 | 1 | 2.5 | 5 | 10 |
| PC-9 | 100 | 101.0758 | 99.38243 | 99.43223 | 90.75636 | 75.24985 | 58.67754 |
| PC-9/GR | 100 | 100.5965 | 94.31528 | 84.89349 | 73.28058 | 65.94644 | 50.2252 |
| PC-9/ER | 100 | 98.8391 | 95.83828 | 91.22753 | 82.00602 | 65.06343 | 45.3281 |

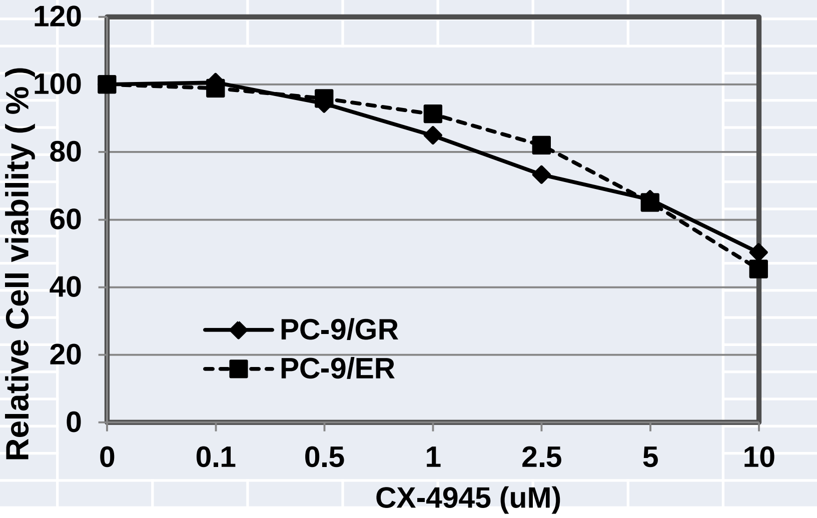


B

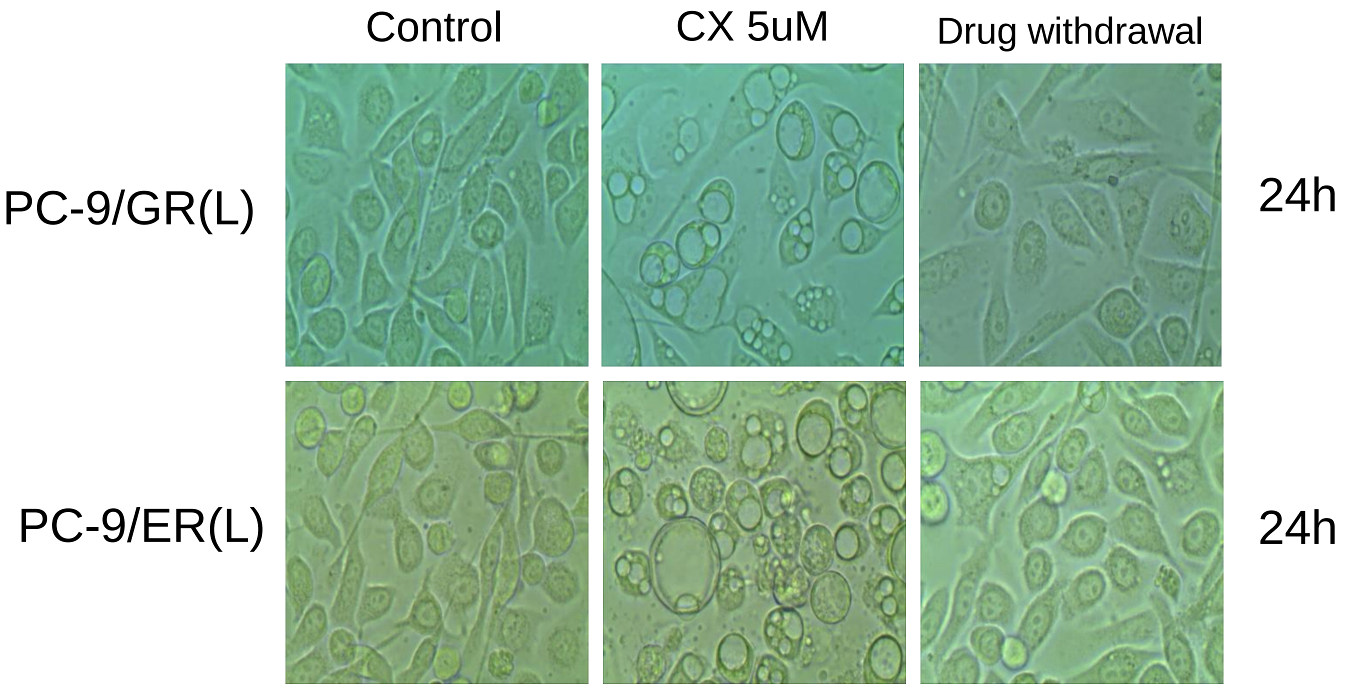

Figure 1

C

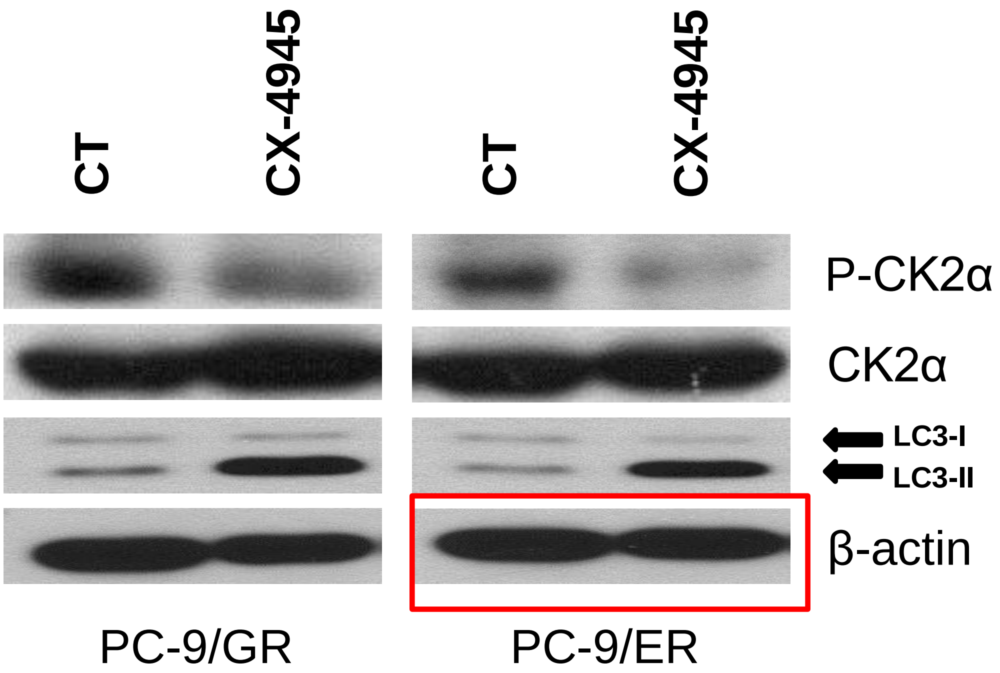


D

140320

PC-9/GR(L), PC-9/ER(L) 2% media, $8x10^5$ cells/60mm Acridine Orange 5ug/ml (20min, 37degree)

CX-4945 5uM, TKI 1uM, combination 24h incubation

| | | CT | CX (5) | G/E (1) | CX (5)+G/E (1) |
|---|---|---|---|---|---|
| PC-9/GR (L) | | 5.45 | 18.5 | 8.5 | 19 |
| PC-9/ER(L) | | 5.15 | 25.1 | 9.35 | 35.25 |
| GR STDEV | | 1.06066 | 2.12132 | 1.838478 | 2.404163056 |
| ER STDEV | | 0.919239 | 3.394113 | 0.636396 | 0.070710678 |
| GR(L) | | 6.2 | 20 | 9.8 | 20.7 |
| | | 4.7 | 17 | 7.2 | 17.3 |
| ER(L) | | 4.5 | 22.7 | 8.9 | 35.3 |
| | | 5.8 | 27.5 | 9.8 | 35.2 |

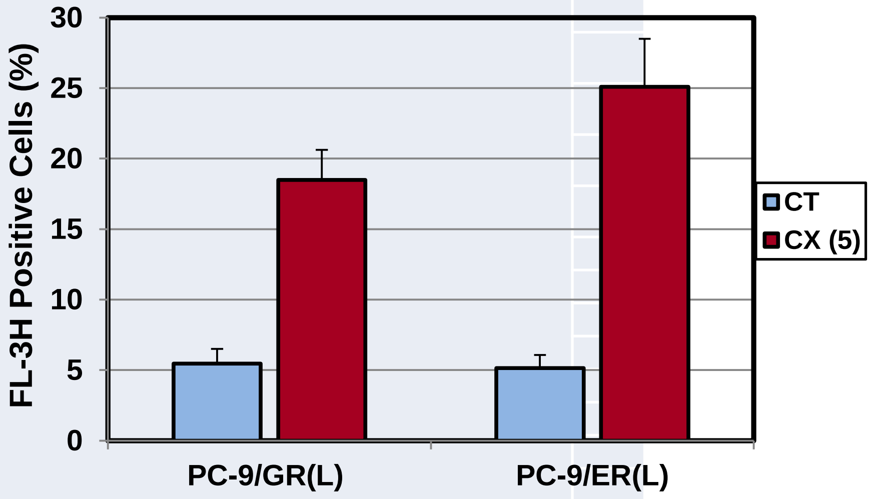

Figure 1

E

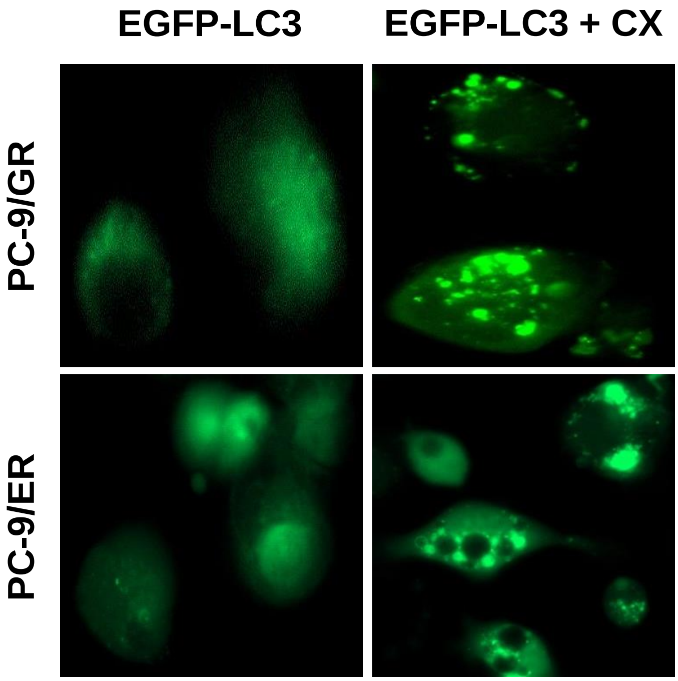
EGFP-LC3
EGFP-LC3 + CX
PC-9/GR
PC-9/ER

F

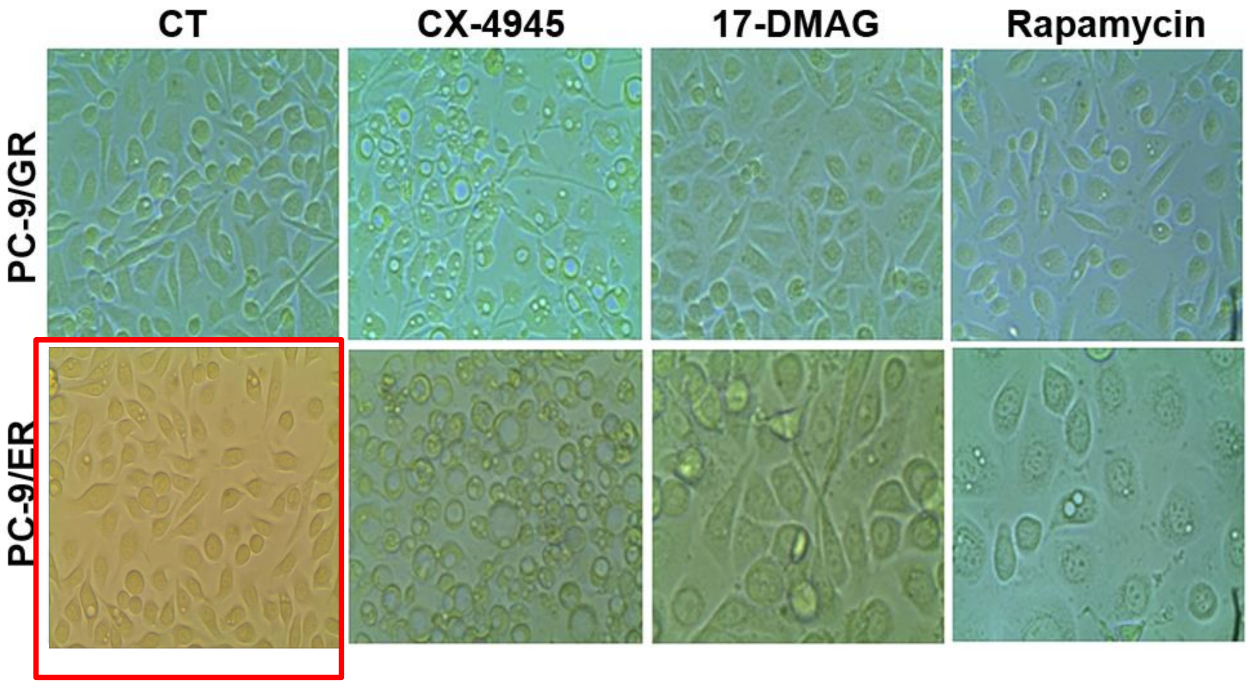
CT
CX-4945
17-DMAG
Rapamycin
PC-9/GR
PC-9/ER

G

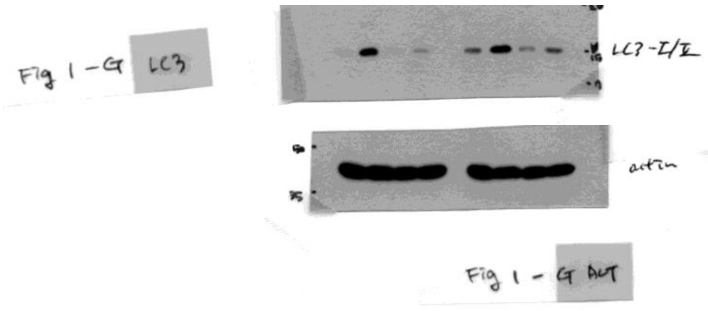
Fig 1-G LC3
LC3-I/II
actin

# Figure 2

## A

| | | | | |
|---|---|---|---|---|
| 140919 | | | | |
| PC-9/GR(L), PC-9/ER(L) 2% media, $1x10^6$ cells/60mm siCK2 100nM | | | | |
| | | | | |
| | Control siRNA | CK2 siRNA | | |
| PC-9/GR | 100 | 68.16609 | | |
| PC-9/ER | 100 | 70.2040816 | | |
| GR STDEV | 5.540762091 | 3.18075715 | | |
| ER STDEV | 5.272287404 | 4.32922519 | | |
| GR(L) | 2680000 | 1840000 | 107.8358 | 63.66782 |
| | 2890000 | 1970000 | 100 | 68.16609 |
| AVE | 2785000 | 1905000 | | |
| ER(L) | 2280000 | 1570000 | 107.4561 | 64.08163 |
| | 2450000 | 1720000 | 100 | 70.20408 |
| AVE | 2365000 | 1645000 | | |

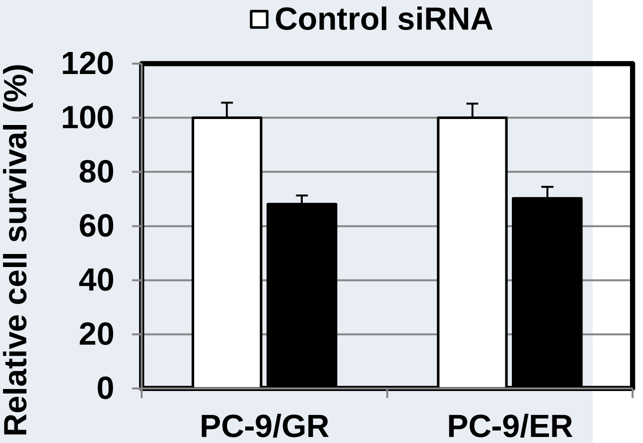


## B

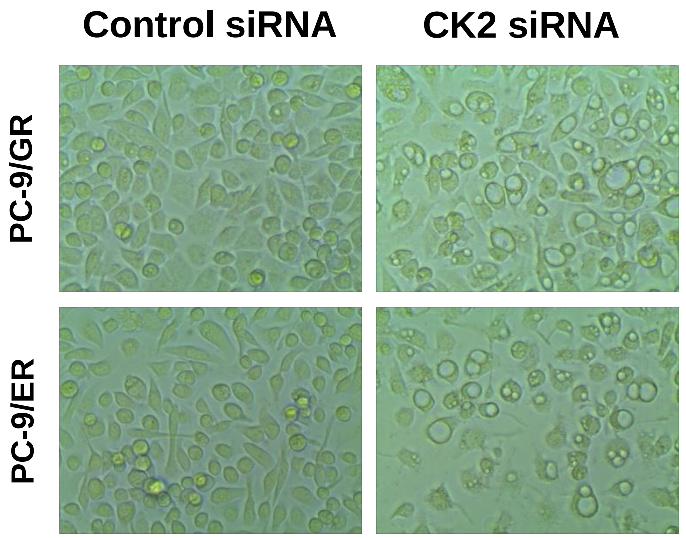

Figure 2

C

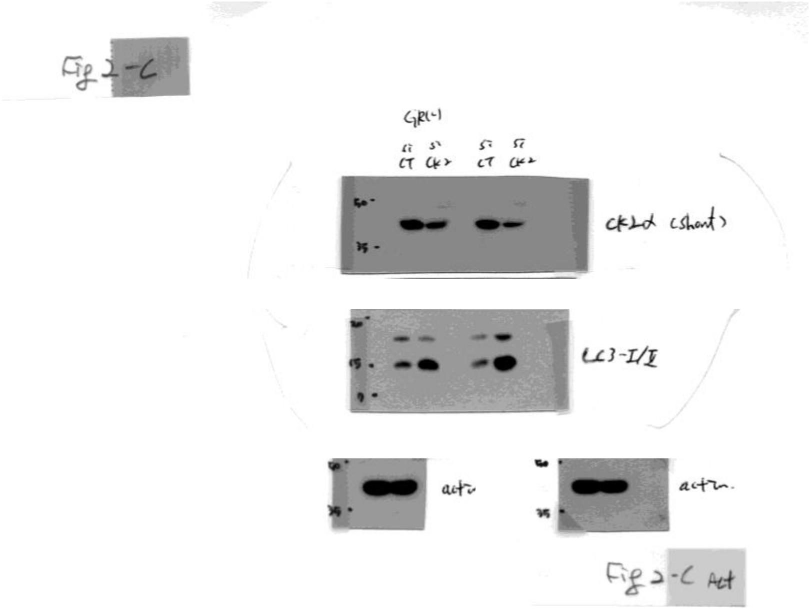

# Figure 3

## A

| | CT | CX (5) | G/E (1) | CX (5)+ G/E (1) |
|---|---|---|---|---|
| PC-9/GR(L) | 100 | 63.63636 | 71.875 | 19.03409091 |
| PC-9/ER(L) | 100 | 61.4786 | 61.8677 | 24.12451362 |
| STEV | 4.662243 | 0.77704 | 1.942601 | 1.165560628 |
| STEV | 7.534744 | 4.636766 | 1.738787 | 1.159191445 |

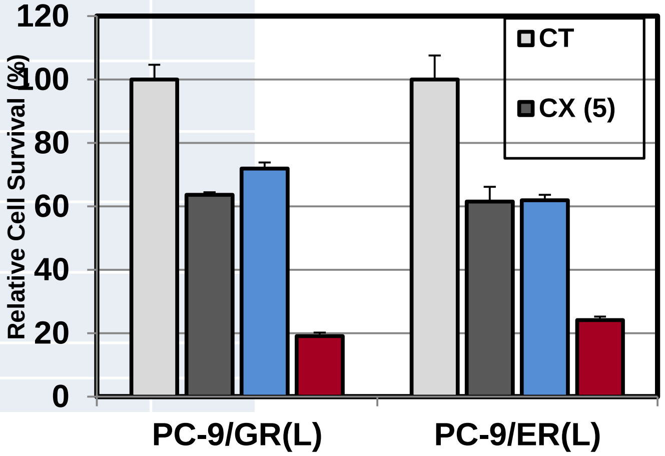


130703
PC-9/GR(L), PC-9/ER(L) 2% media, 8x10[5] cells/60mm Acridine Orange 5ug/ml (20min, 37 degree)
CX-4945 5uM, TKI 0.1, 1uM, combination 48h incubation

| | | CT | CX (5) | G/E (1) | CX (5)+G/E (1) |
|---|---|---|---|---|---|
| | PC-9/GR | 6.3 | 27.7 | 8.6 | 12.7 |
| | PC-9/ER | 5.8 | 37.15 | 16.5 | 16.35 |
| | GR STDEV | 0.565685 | 1.272792 | 1.131371 | 0.848528137 |
| | ER STDEV | 0.565685 | 1.202082 | 1.131371 | 1.48492424 |
| | GR(L) | 6.7 | 26.8 | 9.4 | 13.3 |
| | | 5.9 | 28.6 | 7.8 | 12.1 |
| | ER(L) | 5.4 | 38 | 17.3 | 17.4 |
| | | 6.2 | 36.3 | 15.7 | 15.3 |

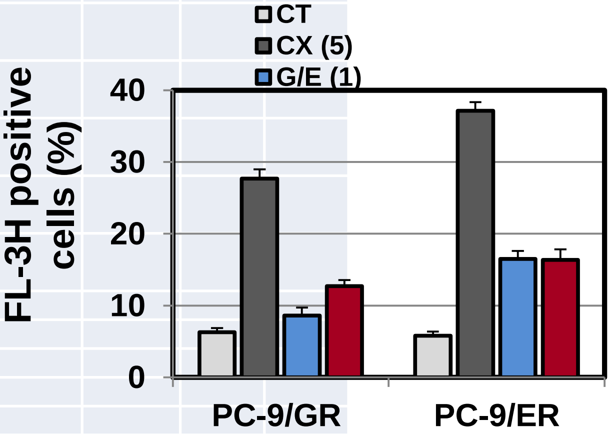

Figure 3

B

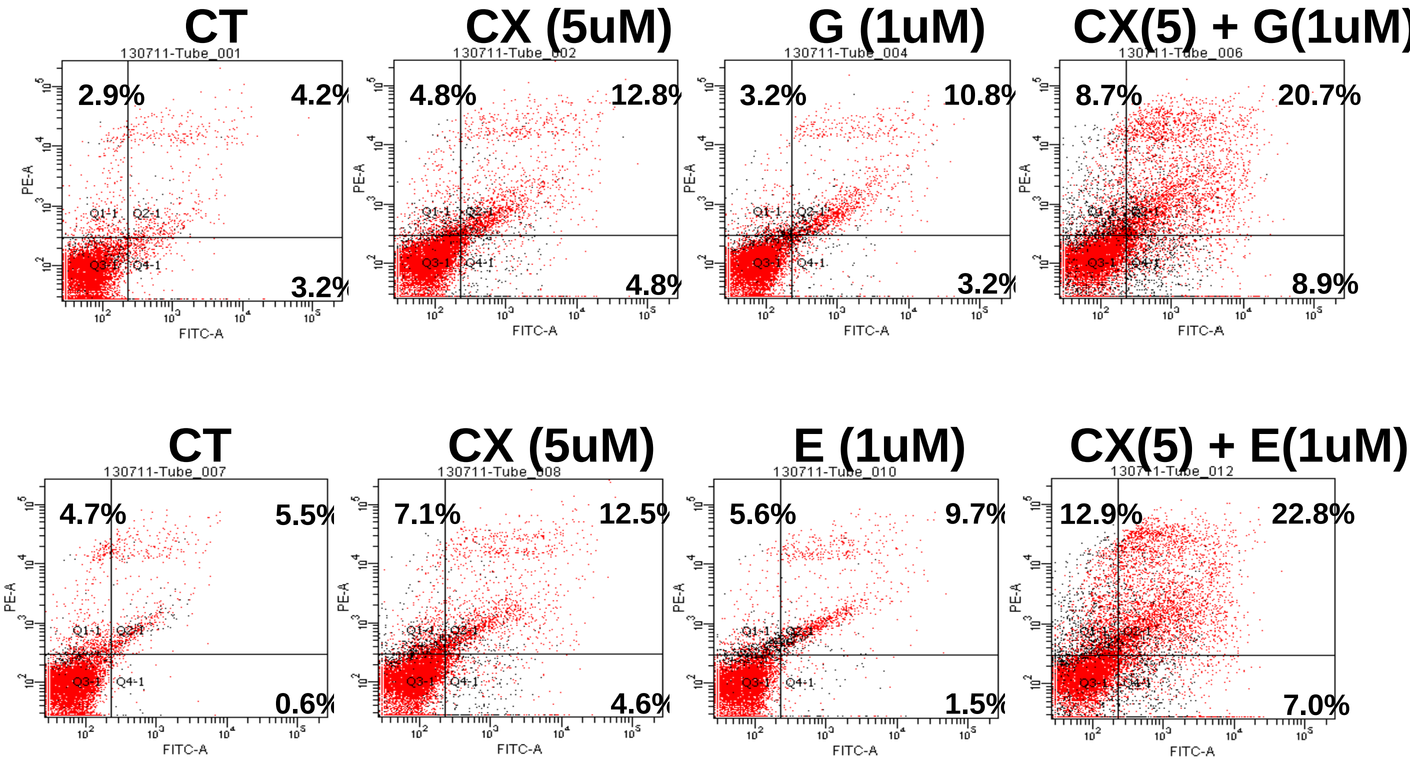


| | | | | |
|---|---|---|---|---|
| 130711 | | | | |
| PC-9/GR(L), PC-9/ER(L) 2% media, 1x10$^6$ cells/60mm | | | | |
| CX-4945 5uM, TKI 0.1, 1uM, combination 48h incubation | | | | |
| | CT | CX (5) | G/E (1) | CX (5)+G/E (1) |
| PC-9/GR | 9.666667 | 18.13333 | 14.8 | 35.4 |
| PC-9/ER | 6.8 | 17.7 | 12.83333 | 38.1 |
| STDEV | 2.250185 | 1.011599 | 0.7 | 5.8 |
| | 0.7 | 0.6 | 1.650253 | 8.3 |

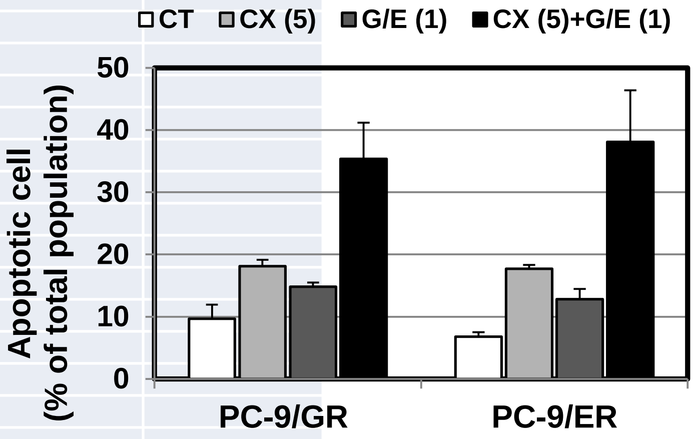

# Figure 3

C

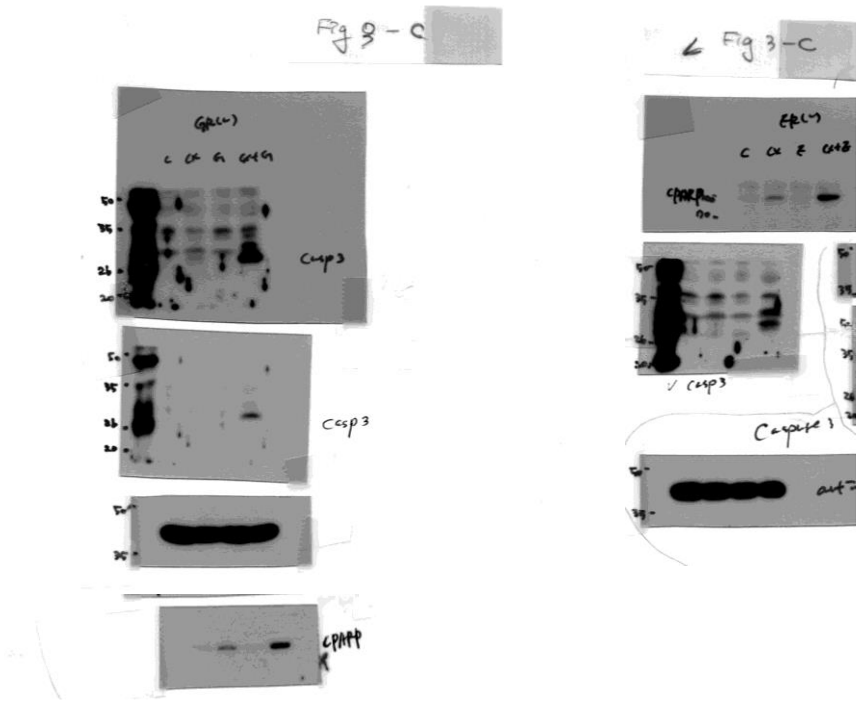

Figure 4

A

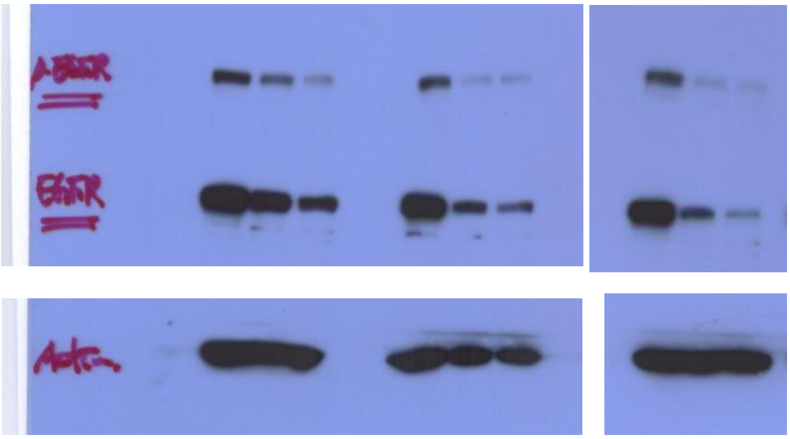

B

| | | | | | | | | | |
|---|---|---|---|---|---|---|---|---|---|
| PC-9 | | | | | | | | | |
| CT | 716 | 100 | 649 | 100 | | 100 | 100 | 100 | |
| EGFR-1 | 173 | 24.162 | 198 | 30.508 | | 24.162 | 30.51 | 27.335 | 4.488 |
| EGFR-2 | 55.4 | 7.7374 | 43.7 | 6.7334 | | 7.7374 | 6.733 | 7.2354 | 0.71 |
| | | | | | | | | | |
| PC-9/GR | | | | | | | | | |
| CT | 798 | 100 | 955 | 100 | | 100 | 100 | 100 | 0 |
| EGFR-1 | 243 | 30.451 | 393 | 41.152 | | 30.451 | 41.15 | 35.801 | 7.567 |
| EGFR-2 | 123 | 15.414 | 133 | 13.927 | | 15.414 | 13.93 | 14.67 | 1.051 |
| | | | | | | | | | |
| PC-9/ER | | | | | | | | | |
| CT | 1000 | 100 | 883 | 100 | | 100 | 100 | 100 | 0 |
| EGFR-1 | 366 | 36.6 | 400 | 45.3 | | 36.6 | 45.3 | 40.95 | 6.152 |
| EGFR-2 | 61.9 | 6.19 | 50.2 | 5.6852 | | 6.19 | 5.685 | 5.9376 | 0.357 |
| | | | | | | | | | |
| | | | 100 | 27.335 | 7 | | 0 | 4.4876 | 0.71 |
| | | | 100 | 35.801 | # | | 0 | 7.5665 | 1.051 |

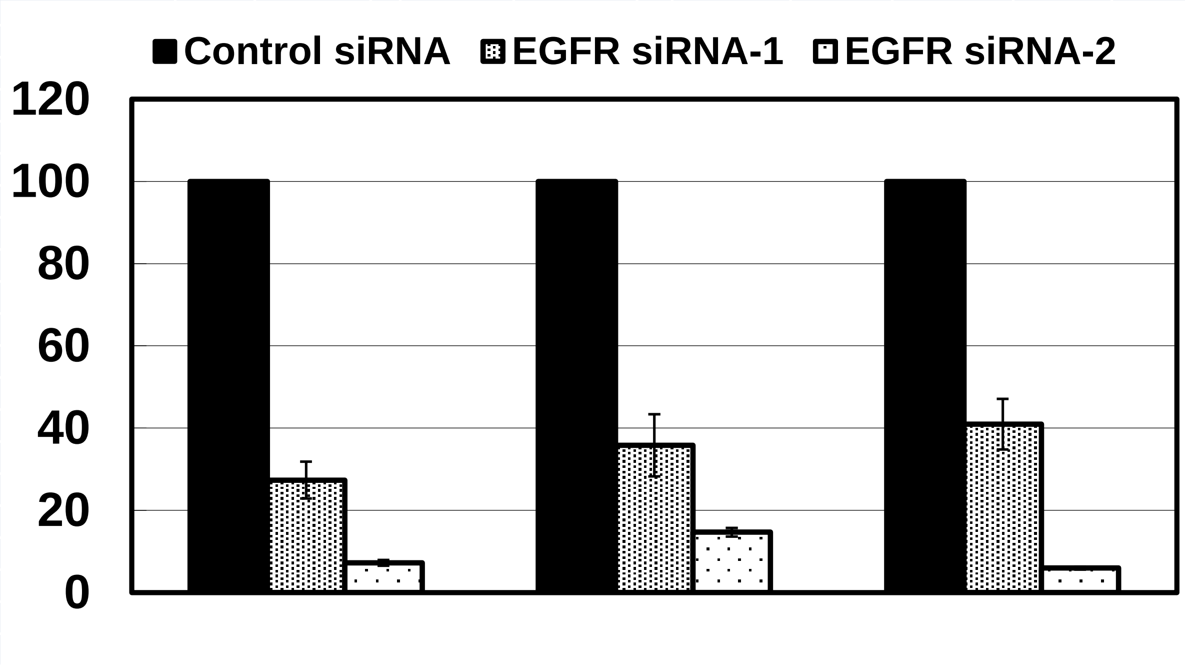

Figure 4

C

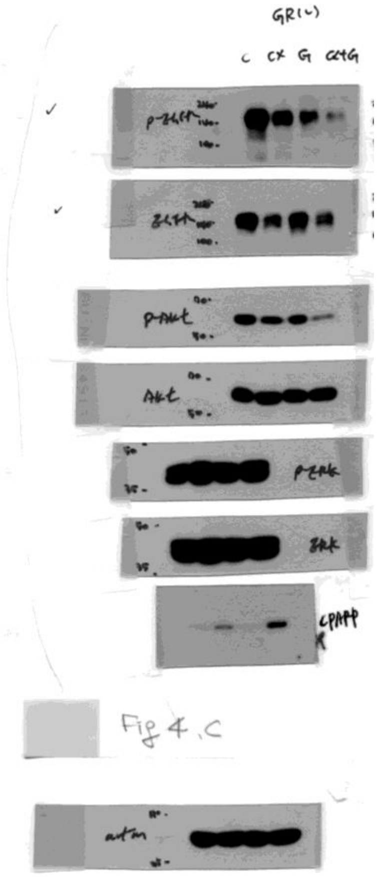


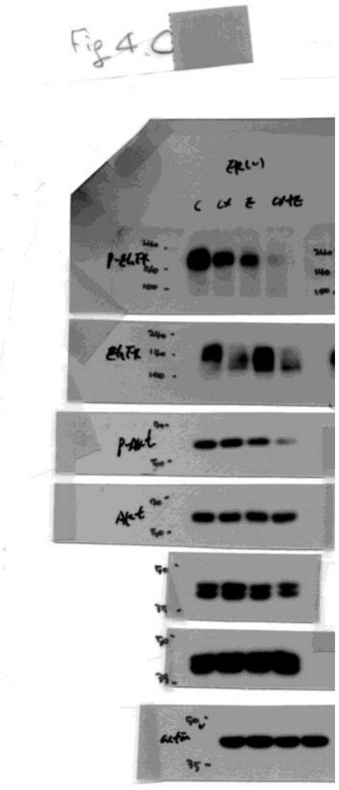

Figure 5

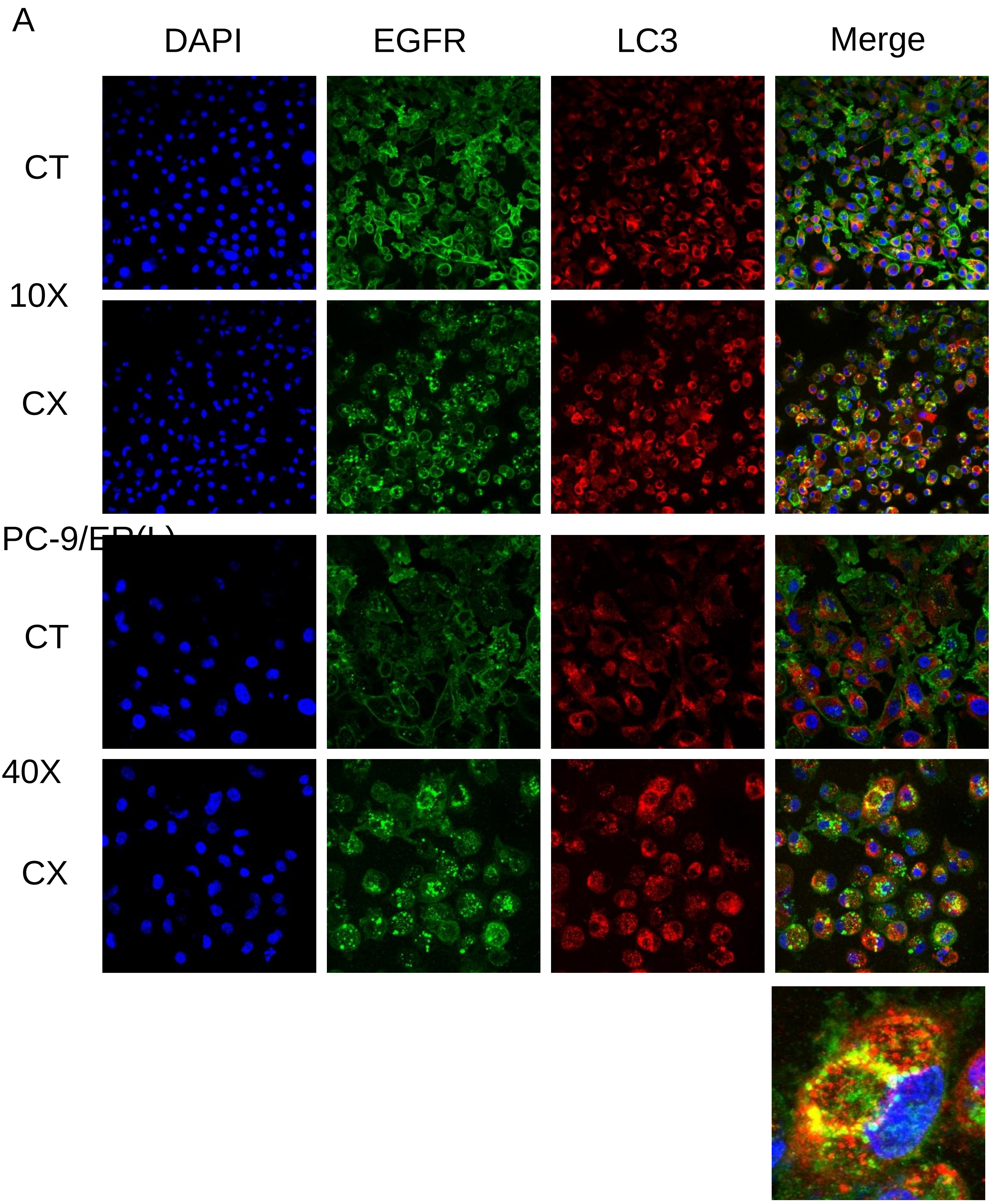

# Figure 5

B

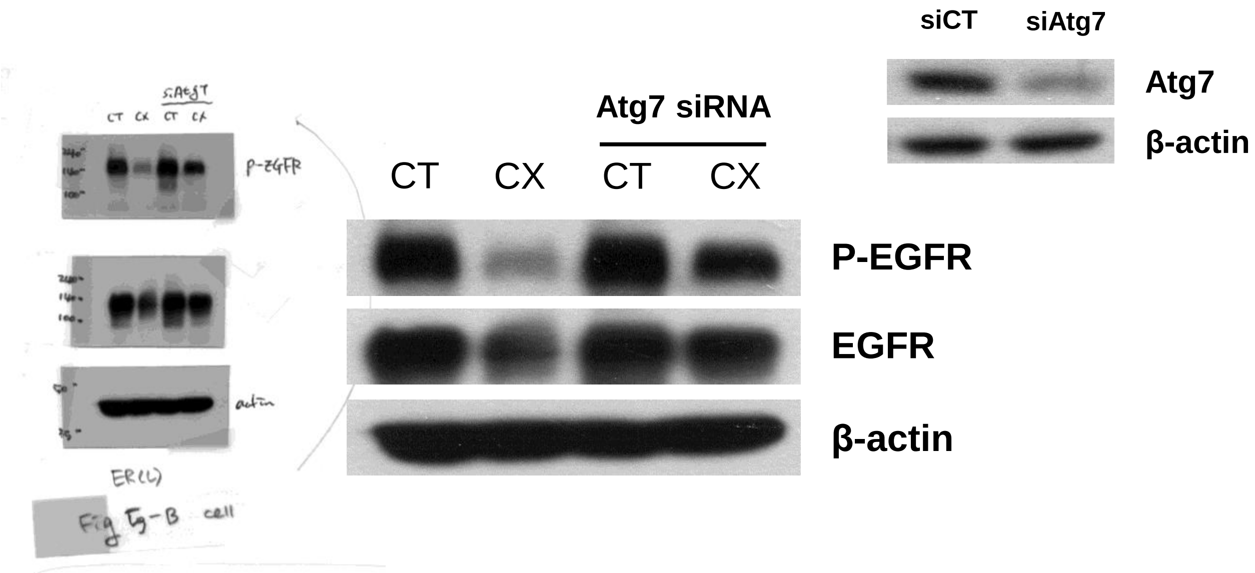
siAtg7
CT CX CT CX
P-EGFR
actin
ER(L)
Fig 5-B cell
Atg7 siRNA
CT
CX
CT
CX
P-EGFR
EGFR
β-actin
siCT
siAtg7
Atg7
β-actin

C

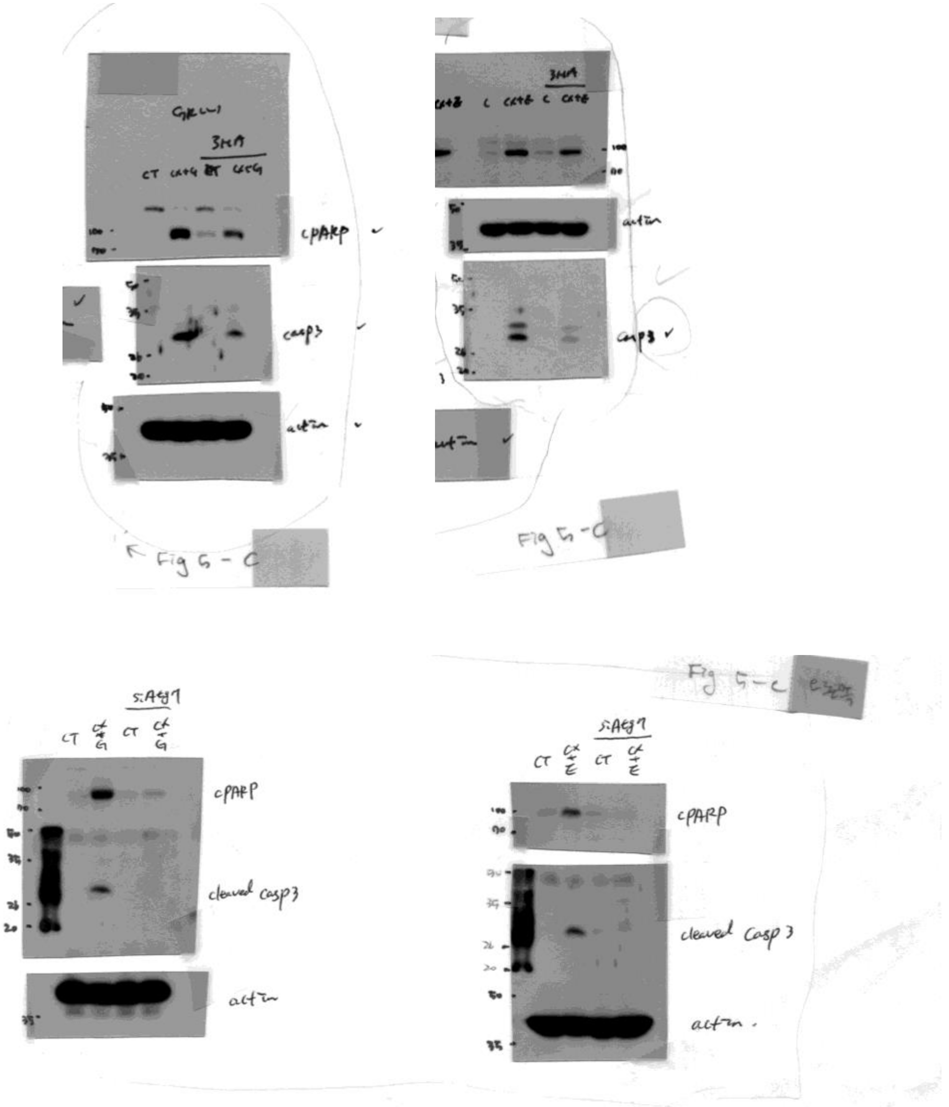
3MA
cPARP
casp3
actin
Fig 5-C
siAtg7
cPARP
cleaved casp3
actin
siAtg7
cPARP
cleaved casp3
actin
Fig 5-C

# Figure 5

## D

140320

PC-9/GR(L), PC-9/ER(L) 2% media, 8x10$^5$ cells/60mm

3MA GR(L) 2mM, ER(L) 1mM 전처리

CX-4945 5uM, TKI 1uM, combination 24h incubation

| | CT | CX+G/E | 3MA | 3MA+CX+G/E |
|---|---|---|---|---|
| PC-9/GR(L) | 4.9 | 20.85 | 8.8 | 11.5 |
| PC-9/ER(L) | 7.45 | 22.5 | 6.85 | 14 |
| GR STDEV | 0.141421 | 0.212132034 | 0.282843 | 0.707106781 |
| ER STDEV | 3.464823 | 2.121320344 | 0.494975 | 0 |
| GR(L) | 4.8 | 21 | 9 | 12 |
| | 5 | 20.7 | 8.6 | 11 |
| ER(L) | 9.9 | 21 | 7.2 | 14 |
| | 5 | 24 | 6.5 | 14 |

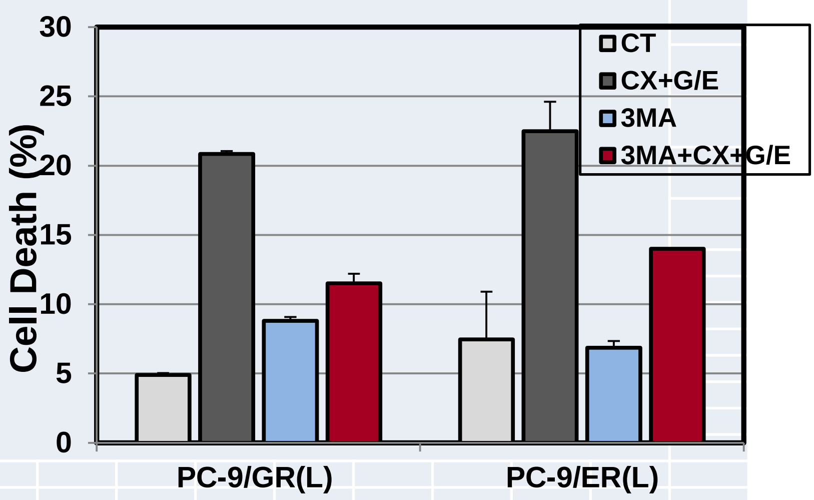


140919

PC-9/GR(L), PC-9/ER(L) 2% media, 1x10$^6$ cells/60mm

Annexin-V/FITC + PI staining

CX-4945 5uM, TKI 0.1, 1uM, combination 48h incubation

siATG7 20nM

| | CT | CX+G/E | siAtg7 | siAtg7+CX+G/E |
|---|---|---|---|---|
| PC-9/GR | 5.75 | 30.55 | 5.55 | 8.9 |
| PC-9/ER | 6.15 | 34.5 | 5.4 | 14.2 |
| GR STDEV | 0.353553 | 3.181981 | 0.494975 | 0.848528137 |
| ER STDEV | 0.919239 | 3.676955 | 0.565685 | 2.2627417 |
| GR(L) | 6 | 32.8 | 5.2 | 8.3 |
| | 5.5 | 28.3 | 5.9 | 9.5 |
| ER(L) | 5.5 | 37.1 | 5 | 15.8 |
| | 6.8 | 31.9 | 5.8 | 12.6 |

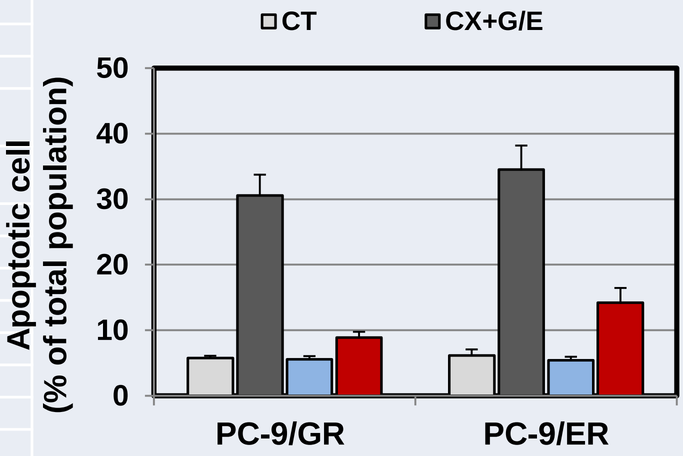

Supplement: S1 File — (PDF) [file pone.0213989.s001.pdf]
